# Surgical treatment of a ventricular aneurysm in a patient with essential thrombocythemia complicated by acute myocardial infarction: A case report

**DOI:** 10.3892/etm.2013.1389

**Published:** 2013-11-07

**Authors:** DONG-WEN WANG, YONG ZHANG, JIAN-MIN YAO, ZHI-BIN XIAO

**Affiliations:** Department of Cardiovascular Surgery, Beijing Military Region General Hospital, Beijing 100700, P.R. China

**Keywords:** essential thrombocythemia, acute myocardial infarction, ventricular aneurysm, surgical treatment

## Abstract

Essential thrombocythemia (ET) is a chronic clonal myeloproliferative disorder, which is often complicated by arterial or venous thrombosis and idiopathic bleeding diathesis. The present study reports a female patient with ET complicated by acute myocardial infarction, leading to ventricular aneurysm following interventional therapy for 3 years and a subsequent in-stent restenosis. Following careful examination, a ventricular aneurysm resection and coronary artery bypass graft were carried out. During this case, the monitoring and controlling of the platelet count, pre- and post-operatively, was extremely important for successful surgery.

## Introduction

Essential thrombocythemia (ET) is a chronic clonal myeloproliferative disorder, which is characterized by persistent thrombocytosis and complicated by arterial or venous thrombosis and idiopathic bleeding diathesis. These complications are the primary causes of mortality in patients with ET (1–3). Although ET-related thrombosis is uncommon in coronary arterial thrombosis, it is more common in extremity vascular thrombosis and may subsequently cause acute myocardial infarction (AMI). Cases of ET complicated with AMI have been reported previously, but have not been treated with surgical therapy (4–6). The present case study reports a patient with ET complicated by AMI, leading to ventricular aneurysm following interventional therapy for 3 years and subsequent in-stent restenosis. Following careful examination, a ventricular aneurysm resection and coronary artery bypass graft were carried out.

## Case report

**Figure 1:**
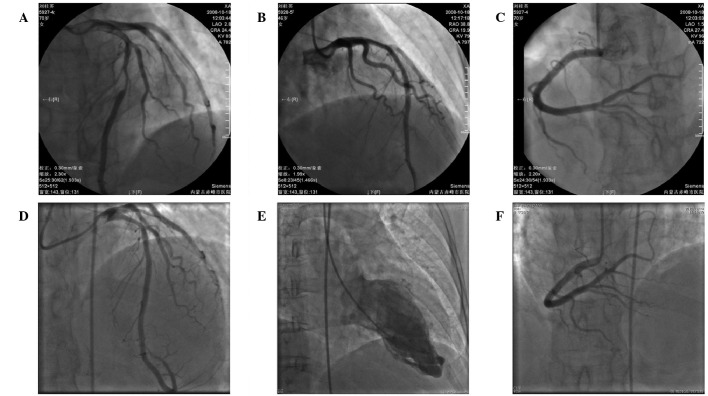
Coronary angiography of an essential thrombocythemia case with acute myocardial infarction. Examination results of the (A–C) first and (D–F) second time admissions.

The present patient was a 48-year-old female who had been admitted to Beijing Tiantan Hospital (Beijing, China) 2 years previously and had complained of a persistently congested chest and radiated pain in her left shoulder for a period of 6 hours. Electrocardiography revealed AMI of the anterior wall. The biomarkers of myocardial injury were significantly increased (troponin I, 12.27 ng/ml; creatine kinase, 1,548 IU/l; and creatine kinase isozyme CK-MB, 189.0 U/l) compared with the normal range (troponin I, <0.04 ng/ml; creatine kinase, 24–170 IU/l; and CK-MB, 0.97–2.88 ng/ml). Laboratory tests revealed that levels of leukocytes (7.1×10^9^/l), hemoglobin (128 g/l), high density lipoprotein (1.20 mmol/l), low density lipoprotein (2.51 mmol/l) and fasting blood-glucose (5.4 mmol/l) were in the normal range. However, platelet levels (8.89×10^11^/l) were higher than the normal range (1–3×10^11^/l). The patient was treated using primary percutaneous coronary intervention. Intraoperative observations indicated that 90% of the near front descending branch was narrowed and the remainder of the aortic wall was smoothed. Subsequently, a rapamycin-eluting stent (3.5×29 mm) was embedded in the front descending branch (Fig. 1A, B and C). Postoperatively, chest congestion was relieved and the patient was discharged. The patient was initially administered with clopidogrel (75 mg per day) to reduce the platelet count, which was subsequently replaced with enteric aspirin 1 year later (100 mg per day).

The patient complained of palpitations and shortness of breath one month prior to the second admission. During this period, the patient experienced paroxysmal nocturnal dyspnea and was unable to lie in a horizontal position at night. High blood pressure, diabetes or hyperlipoidemia were not observed or a history of smoking. The patient had been diagnosed with ET in the Beijing Tiantan Hospital 15 years previously. All routine test references were within the normal range, with the exception of the platelet count (5.52×10^11^/l). Color doppler echocardiography was performed and identified a ventricular aneurysm. Furthermore, coronary arteriography revealed a 50% narrowing in the front descending stent and the formation of a left ventricular aneurysm (Fig. 1D and E). The symptoms of cardiac insufficiency disappeared following symptomatic treatment. The patient was administered hydroxyurea (100 mg per day) for a week in order to reduce platelet count to 325×10^9^/l. The ventricular aneurysm was resected under general anesthesia and cardiopulmonary bypass. Bypass surgery was performed between the left internal mammary artery and the front descending stent. The patient recovered well and no complications, for example hemorrhage or thrombosis, occurred. The platelet count was controlled using hydroxyurea (100 mg per day) and aspirin (100 mg per day). At ~15 days following surgery, the platelet count was 286×10^9^/l and the patient was discharged. At the 3-month follow-up, the patient showed no signs of heart insufficiency or angina. An ultrasonic cardiogram showed that the left ventricular aneurysm had disappeared. Furthermore, no heart-related symptoms were detected at a 2-year follow-up. The study was approved by The Ethics Committee of Being Military Region General Hospital, Beijing, China. Written informed patient consent was obtained from the patient or the patient’s family.

## Discussion

The annual incidence of ET is ~1.5 per 100,000 individuals worldwide (7). The principal causes of morbidity and mortality in ET are thrombosis, hemorrhage and progression to myelofibrosis or acute myelogenous leukemia (8). Only 9.4% of patients with ET have myocardial infarction (9). Although the median age of patients with ET is 60 years, 10–25% of patients are <40 years of age and one-third of patients are asymptomatic (10–13). With the exception of a history of thrombosis, cardiovascular disease risk factors, including smoking, high blood pressure, hyperlipidemia and diabetes, are also risk factors for thrombosis (12).

In the present report, we describe an ET case complicated with AMI and the formation of a ventricular aneurysm. Preoperative examinations showed no cardiovascular disease risk factors and the vital organs were healthy. Therefore, a history of ET was the only surgical risk. Although the incidence rate of thrombosis is higher than bleeding for ET cases, the risk of bleeding is relatively higher in surgical cases (14). Regardless of the complications of thrombosis or hemorrhage, the monitoring and control of the platelet count, preoperatively or postoperatively, is key for a successful surgery. In this case, the patient maintained a relatively normal platelet count as a result of treatment with hydroxyurea and the platelet count in the perioperative period was consistent with previous case reports (15–17). Meanwhile, such alteration of the platelet count may also be controlled postoperatively.

Similar cases have been reported in previous studies. However, the majority of such cases were treated with an interventional approach and rarely via surgical methods. ET, complicated by coronary artery thrombosis, has no uniform treatment guidelines worldwide and therefore clinical treatment is difficult. As well as utilizing anti-platelet drugs, including aspirin and clopidogrel, it is important to use platelet-cytoreductive therapy for ET patients (18–20). In the case of coronary artery stent restenosis, we recommend a combined treatment of hydroxyurea and aspirin to prevent thrombosis.

In conclusion, surgical treatment for ET with myocardial infarction is rare and no uniform treatment guidelines are currently available worldwide. Therefore, ET is a key risk in surgery. It is important to prevent thrombosis and hemorrhage, as well as monitor and control the platelet count preoperatively and postoperatively in order to achieve a successful surgery.
